# Higher BMI, but not obesity-related genetic polymorphisms, correlates with lower structural connectivity of the reward network in a population-based study

**DOI:** 10.1038/s41366-020-00702-4

**Published:** 2020-10-25

**Authors:** Frauke Beyer, Rui Zhang, Markus Scholz, Kerstin Wirkner, Markus Loeffler, Michael Stumvoll, Arno Villringer, A. Veronica Witte

**Affiliations:** 1 Department of Neurology, Max Planck Institute for Human Cognitive and Brain Sciences, 04103 Leipzig, Germany; 2 Collaborative Research Centre 1052 ’Obesity Mechanisms’, Subproject A1, Faculty of Medicine, Leipzig University, 04103 Leipzig, Germany; 3 Faculty of Medicine, Institute for Medical Informatics, Statistics and Epidemiology (IMISE), Leipzig University, 04103 Leipzig, Germany; 4 Leipzig Research Center for Civilization Diseases (LIFE), Leipzig University, 04103 Leipzig, Germany; 5 Department of Endocrinology and Nephrology, Leipzig University Hospital, 04103 Leipzig, Germany; 6 Clinic of Cognitive Neurology, Leipzig University Hospital, 04103 Leipzig, Germany

**Keywords:** Neuroscience, Epidemiology

## Abstract

**Background:**

Obesity is of complex origin, involving genetic and neurobehavioral factors. Genetic polymorphisms may increase the risk for developing obesity by modulating dopamine-dependent behaviors, such as reward processing. Yet, few studies have investigated the association of obesity, related genetic variants, and structural connectivity of the dopaminergic reward network.

**Methods:**

We analyzed 347 participants (age range: 20–59 years, BMI range: 17–38 kg/m^2^) of the LIFE-Adult Study. Genotyping for the single nucleotid polymorphisms rs1558902 (FTO) and rs1800497 (near dopamine D2 receptor) was performed on a microarray. Structural connectivity of the reward network was derived from diffusion-weighted magnetic resonance imaging at 3 T using deterministic tractography of Freesurfer-derived regions of interest. Using graph metrics, we extracted summary measures of clustering coefficient and connectivity strength between frontal and striatal brain regions. We used linear models to test the association of BMI, risk alleles of both variants, and reward network connectivity.

**Results:**

Higher BMI was significantly associated with lower connectivity strength for number of streamlines (*β* = −0.0025, 95%—C.I.: [−0.004, −0.0008], *p* = 0.0042), and, to lesser degree, fractional anisotropy (*β* = −0.0009, 95%—C.I. [−0.0016, −0.00008], *p* = 0.031), but not clustering coefficient. Strongest associations were found for left putamen, right accumbens, and right lateral orbitofrontal cortex. As expected, the polymorphism rs1558902 in FTO was associated with higher BMI (*F* = 6.9, *p* < 0.001). None of the genetic variants was associated with reward network structural connectivity.

**Conclusions:**

Here, we provide evidence that higher BMI correlates with lower reward network structural connectivity. This result is in line with previous findings of obesity-related decline in white matter microstructure. We did not observe an association of variants in FTO or near DRD2 receptor with reward network structural connectivity in this population-based cohort with a wide range of BMI and age. Future research should further investigate the link between genetics, obesity and fronto-striatal structural connectivity.

## Introduction

Obesity or excess body weight is the result of an imbalance in energy intake and expenditure and is now mainly considered a neurobehavioral disorder, which involves homeostatic brain regions as well as regions engaged in salience and reward processing [1]. Although increasing obesity rates in western societies may be primarily driven by the availability of high-fat diets and a sedentary lifestyle [2], the risk of developing obesity is under strong (polygenetic) control, with heritability estimates of 50–70% [3, 4]. Yet, it is poorly understood which individual (genetic) predispositions determine the vulnerability to those environmental influences and how they lead to excessive weight gain and obesity.

Single nucleotid polymorphisms in the fat- and obesity-related gene (FTO) are the most common genetic variants associated with obesity [5]. Yet, little is known about the mechanisms underlying this association [6]. Variation in FTO may contribute to the risk for obesity by modulating feeding behavior rather than energy expenditure [7]. Here, one possible mechanism is that polymorphisms in FTO influence dopamine signaling between the nucleus accumbens (Nacc) and frontal brain regions, either directly or by interaction with dopamine-related variants [8]. Consequently, behavioral changes in reward processing, learning and impulsivity might lead to differences in eating behavior, and ultimately, weight gain [9–12].

Functional neuroimaging studies have provided evidence for a main effect of FTO on the neural response to food cues, and reported that this association depended on fasting state and ghrelin signaling [13–17]. Further studies have shown that FTO interacted with the Taq1A polymorphism, located near the D2 dopamine receptor (DRD2), to modulate reward learning and neural activity in response to food cues—both key functions of the Nacc [18–20].

Besides functional activation, the strength of structural connections between the Nacc and frontal brain regions plays an important role for dopamine-dependent behavior [21, 22]. Van Schouwenburg and colleagues showed that the effects of a dopaminergic drug depended on fronto-striatal structural connectivity [23]. Previously, individual effects of dopaminergic drugs have been related to baseline working memory capacity, which presumably reflects baseline dopamine levels [24]. Along these lines, a DRD2 variant (rs6277 C-allele), previously linked to reduced striatal binding potential, was associated with increased fronto-striatal structural connectivity [25]. This variant is in linkage disequilibrium with the Taq1A (*D*′ = 0.66) [26].

Regarding the structural connectivity of the reward network in obesity, studies showed contradicting results, with both higher and lower structural connectivity between striatum and frontal cortex associated with weight status [27–29]. For other white matter tracts, studies have consistently reported associations of higher body mass index (BMI) and lower WM microstructure, possibly mediated by the negative metabolic impact of obesity [30, 31]. Recently, a variant in FTO was shown to interact with weight status on fronto-striatal structural connectivity, indicating that some of the mixed results might be due to genetic variability or differences in tonic dopamine levels [32, 33].

In summary, while there is consistent evidence of obesity-related differences in global white matter microstructure, less is known about the association of BMI and reward network white matter structure, and the impact of obesity-related genotypes on this trait. In this study, we aimed to investigate the association of structural reward network connectivity, obesity, and genetic variations linked to obesity in a well-characterized population-based sample. Based on previous studies, we hypothesized that a higher BMI would relate to lower reward network connectivity [27]. More exploratory, we investigated whether FTO and Taq1A polymorphisms had interactive or independent effects on structural connectivity within the reward network [19, 32]. This may help to better understand the genetic and neurobehavioral background of obesity.

## Methods

### Participants

All participants took part in the MRI assessment of the LIFE-Adult-Study (*n* ~ 2600) [34]. The study was approved by the institutional ethics board of the Medical Faculty of the University of Leipzig and conducted according to the declaration of Helsinki. Besides age and sex stratification, participants were randomly selected from the registry of the city of Leipzig and gave written informed consent. We included participants aged between 20 and 59 years (*n* ~ 800) with complete information on BMI and genotyping (*n* ~ 400). Further, we excluded participants with stroke [1], neuroradiological findings of brain pathology [11], cancer treatment in the last 12 months [5], epilepsy [5], multiple sclerosis [1], and intake of centrally active medication (*n* = 25).

Due to incomplete fat suppression during the diffusion-weighted imaging (DWI) acquisition, an artifactual rim in the parietal lobe of the brain was present in many DWI scans. As this artifact was difficult to detect in an automated fashion, we manually rated all scans into three categories dependent on the number and severity of affected slices, i.e., “no/very mild”, “moderate”, and “severe”. We restricted our analysis to those scans that were rated as “no/very mild” and “moderate”. Two participants were excluded due to outlying values in structural connectivity (see connectome reconstruction from DWI data). This led to a total sample of 347 healthy volunteers eligible for analysis. We did not perform a formal power analysis before the study. Yet, we estimate our power to be sufficient (*β* = 0.98, based on *N* = 150 in the under- and normal-weight group, *N* = 197 in the overweight and obese group, alpha = 0.05 and the lowest Cohen’s *d* reported in ref. [27] of *d* = 0.44 for FA CC difference, calculated using ‘pwr’ in R version 3.6.1).

### Genotyping

Genomic DNA was extracted from EDTA-treated blood samples using the Autopure LS instrument (Qiagen, Hilden). Genotyping of FTO polymorphism rs1558902 and rs1800497 (Taq1A), a polymorphism near dopamine D2 receptor, was performed based on using the Affymetrix Axiom Technology with custom option (Axiom-CADLIFE). Individuals were imputed at the 1000Genomes reference phase 1, release 3 (http://mathgen.stats.ox.ac.uk/impute/data_download_1000G_phase1_integrated.html) using SHAPEIT v2 and IMPUTE 2.3.0. Details of the measurement and quality control can be found elsewhere [35].

We selected the rs1558902 polymorphism on FTO based on a large BMI meta-GWAS [36]. The variant is in high linkage disequilibrium with other obesity-associated SNPs, e.g., rs9939609. The risk allele of this variant is the A allele and the genotype frequencies in our sample were TT *n* = 116, AT *n* = 170, AA *n* = 61 (minor allele frequency = 0.42, test for Hardy–Weinberg equilibrium Chi^2^ = 0.0008, df = 0.97). We considered three groups of risk carriers for the analysis (0: TT, 1: AT, 2: AA).

For the rs1800497 polymorphism, the genotype frequencies were TT *n* = 249, AT *n* = 89, AA *n* = 9 (minor allele frequency = 0.15, test for Hardy–Weinberg equilibrium Chi^2^ = 0.02, df = 1, *p* = 0.89). We grouped together carriers of at least one risk allele (A).

### Anthropometric measurement

Anthropometric measurements were conducted by trained study personal. Body weight was measured with scale SECA 701, height was measured with height rod SECA 220 (SECA Gmbh & Co. KG). BMI was calculated as the weight in kilograms divided by the square height in meters (kg/m^2^).

### Possible confounders

We performed sensitivity analysis of our results by considering different confounders. Head motion is an important confounder in neuroimaging studies, not only affecting measures of functional connectivity, but also measures of brain structure. Since we previously noticed a strong collinearity between BMI and head motion during resting state, we adjusted for head motion in our analysis of structural connectivity [37]. Therefore, we used the six rotation and translation parameters returned by eddy_correct during preprocessing to calculate mean framewise displacement as described by [38]. Mean FD was used to adjust for head motion during the DWI scan.

Smoking status and education were derived from self-report questionnaires. Smoking status was available for *N* = 337 participants in three levels (0 = never smoker, 1 = previous smoker, 2 = current smoker). Education was given in 3 levels (1 = left school without degree or finished secondary school after 9 years, 2 = finished secondary school after 10 years, 3 = finished secondary school after 12 or 13 years) and was available for *N* = 345 participants. Depression scores were derived from the CES-D questionnaire as previously described and were available in *N* = 326 participants [39]. We log-transformed mean FD and CES-D score prior to the analysis because of the skewed distribution of these scores.

### MR data acquisition

Brain imaging was performed on a 3T Verio Scanner (Siemens, Erlangen) with a 32 channel head coil. T1-weighted images were acquired using generalized autocalibrating partially parallel acquisition imaging technique [40] and the Alzheimer’s Disease Neuroimaging Initiative standard protocol with the following parameters: inversion time, 900 ms; repetition time, 2300 ms; echo time, 2.98 ms; flip angle, 9°; band width, 240 Hz/pixel; image matrix, 256 × 240; 176 partitions; field of view, 256 × 240 × 176 mm^3^; sagittal orientation; voxel size, 1 × 1 × 1 mm^3^; no interpolation.

DWI was performed using a double-spin echo sequence with the following parameters: TR, 13.8 s; TE, 100 ms; image matrix, 128 × 128 × 72; field of view, 220 × 220 × 123 mm^3^, voxel size of 1.7-mm isotropic, max *b* value = 1000 s/mm^2^, 60 directions.

All MRI scans were acquired between 8 a.m. and 2 p.m., rarely at 3 p.m. Participants were randomly assigned to scan slots. Accordingly, age, sex, and BMI were not linearly associated with scanning hour (*n* = 236, correlations and *t*-tests, respectively, all *p* > 0.05). Moreover, structural connectivity measures were not linearly associated with scanning hour (available in *n* = 236; bivariate correlations, all *p* > 0.05). We also did not find evidence for periodic associations with time of day by visually checking the plots. Participants received a small non-standardized breakfast before the scan.

### T1-data processing

Cortical reconstruction and volumetric segmentation of T1-weighted MR images were performed with the Freesurfer image analysis suite (version 5.3.0) [41, 42]. Regions of interest from subcortical segmentation and cortical Desikan-Killiany parcellation were used as nodes in connectome reconstruction. Volumetric measures of reward network regions (bilateral, lateral, and medial orbitofrontal cortex; caudate; putamen; and accumbens) were averaged across hemispheres for analysis.

### Connectome reconstruction from DWI data

Connectome reconstruction was performed during the 10kin1day-workshop, a collaborative science event where over 10000 DWI datasets were processed in a joint effort [43]. Preprocessing included correction for susceptibility and eddy currents using FSL’s tool eddy-correct. Freesurfer’s standard Desikan-Killiany parcellation was used to select 68 cortical and 14 subcortical regions for connectome reconstruction. A diffusion tensor was fitted to each voxel in a white matter mask using robust tensor fitting, and deterministic pathway tractography was applied to construct white matter fiber tracts using a customized script described in [43]. Tracts were started in each voxel and then followed the main diffusion direction using the fiber assignment by continuous tracking algorithm. Stopping criteria were FA value below 0.1, crossing of the brain mask, or a fiber turn of more than 45°. Weighted, unsigned, and undirected connectivity matrices were constructed for each subject by using 82 (sub)cortical regions as nodes and connectivity weights between the regions as edges. Two types of connectivity weights were assessed (1) total number of connecting streamlines touching both regions (NOS) and (2) mean FA across voxels included in these streamlines (FA).

Reward connectivity networks were reconstructed by using bilateral lateral and medial orbitofrontal cortex, caudate, putamen, and accumbens as nodes and their respective structural (NOS and FA strength) connectivity strength (CS) as edges [27]. Data quality assurance was done according to the 10kin1day-workshop guidelines by identifying subjects with outlying values in mean NOS or mean FA connectivity of existing connections or outlying values in prevalence of existing or non-existing connections [43]. Outliers were defined as values higher than (3rd quartile + 2*interquartile range (IQR)) or lower than (1st quartile − 2*IQR). This led to the exclusion of two participants with bad data quality (excessive head motion and large ventricles).

### Reward network analyses

We used a graph theoretical approach to investigate the strength and organization of the reward network. This approach is based on the evidence that large-scale brain networks are intrinsically organized like graphs and show corresponding properties [44]. Following [27], we defined the reward network based on ten bilateral regions (lateral and medial orbitofrontal cortex, caudate, putamen, and accumbens) as nodes and NOS and average FA between regions as edges. CS within the network was assessed as the average connectivity between all pairwise regions. CS indicates the overall strength of connections between these brain regions, either quantitatively (NOS) or qualitatively (FA). Further, we calculated the clustering coefficient (CC) as the average of the CC of the individual nodes. This measure reflects local connectivity, i.e., how densely neighboring nodes in the network are connected to each other. CS and CC measure different aspects of network organization, e.g., overall strength and local organization, and might therefore be differentially associated with our measures of interest.

We calculated the graph metrics (mean FA and mean NOS CC and CS) using the Brain Connectivity Toolbox in Matlab 9.7 (2019b). Then, we normalized the measures by the corresponding whole brain connectivity metrics, resulting in the following reward network metrics: FA CS, FA CC, NOS CS, and NOS CC. The normalization was performed following [27] and aimed to make the result more specific to differences in the reward network.

The NOS structural connectivity weights were normalized to [0, 1] prior to calculating the weighted CC.

For sensitivity analysis, we assessed normalized node-wise CS and CC for the ten nodes (bilateral, lateral, and medial orbitofrontal cortex; caudate; putamen; and accumbens) in the network.

### Statistical analysis

We used univariate linear models in R version 3.6.1 to investigate the association of the BMI, genetic variants, and measures of reward network (REW) structural connectivity [45].

We checked the assumptions of linear regression (normality and homoscedasticity of residuals) visually, and excluded no data point in the main analysis, as these were met. Yet, we excluded moderate outliers in BMI (1.5 IQR above 3rd quartile) in a sensitivity analysis. We use F-tests and associated *p* values from R’s anova to compare full versus null models.

First, we tested the association of BMI and the four reward network measures, adjusting for age and sex (Model 1: REW metric ~ age + sex + BMI). If there was a significant association of BMI, tested by comparing this full model against a null model including only age and sex (Bonferroni-corrected alpha = 0.0125), we additionally adjusted for average head motion during the DWI scan, smoking status, depression, and education (Model 2: REW metric ~ age + sex + head motion + smoking status + depression + education + BMI).

We further investigated the node-wise connectivity if the edge type (i.e., FA or NOS) reached network-wide significance. Thereby we aimed to identify nodes that contributed most to the result. Here, we fitted Model 2 (node-wise REW metric ~ age + sex + head motion + smoking status + depression + education + BMI) and used Bonferroni-corrected alpha = 0.005 (accounting for ten regions of interest tested).

In the genetic analysis, we first investigated whether the polymorphisms rs1558902 and rs180047 explained variance in BMI. We compared a full model including the interaction of rs1558902 (0/1/2 risk-allele groups) with rs180047 (0/1 or 2 risk-allele groups) (Model 3: BMI ~ age + sex + FTO*Taq1A) against a null model without the interaction (Model 4: BMI ~ age + sex + FTO + Taq1A). Without a significant interaction (Chi-square test of Model 3 vs. 4: *p* < 0.05), we report only main effects of model 4.

Then, we tested whether the polymorphisms were associated with reward network connectivity, either independently or interacting with each other. Again, we first tested the interaction effect of the two variants (Model 5: REW metric ~ age + sex + FTO*Taq1A) by comparing it to a null model without the interaction. If there was no indication for an interaction (*p* > 0.05), we checked for independent effects of the two genotypes separately (Model 6: REW metric ~ age + sex + FTO + Taq1A), again by comparing against a null model including age, sex. If we found a significant effect (*p* < 0.05) for any of the two variants, we additionally included head motion, smoking status, depression, education as covariates.

## Results

In total, we analyzed 347 participants (53% women), for descriptive statistics see Table 1.

**Table 1:** Demographic characteristics of the sample (*n* = 378, sex distribution: 170 men and 208 women).

	*n*	Minimum	Maximum	Mean	Std. deviation
Age [years]	347	20	59	45.7	9.0
BMI [kg/m^2^]	347	17.7	38.2	25.7	3.6
Weight groups (underweight/normal weight/overweight/obese)	347	2/148/159/38			
FTO rs1558902 polymorphism (TT/AT/AA)	347	116/170/61			
Near DRD2 Taq1A polymorphism rs1800497 (0/at least 1 risk allele)	347	249/98			
Depression scores (CES-D)	326	0	46	9.5	6.8
Smoking status (never smoker/previous smoker/current smoker)	337	175/79/83			
Education (no degree or 9/10/10/12–13 years)	345	9/190/146			
Mean FD	347	0.4	1.3	0.62	0.13
Fat suppression artifact in DWI data (“no/very mild”/“moderate”)	347	318/29			

Weight groups according to WHO definition (underweight: BMI < 18 kg/m^2^, normal weight: ≥18, <25 kg/m^2^, overweight: ≥25, <30 kg/m^2^, obese: ≥30 kg/m^2^).

*BMI* body mass index, *CES-D* Center for Epidemiologic Studies Depression Scale, *mean FD* mean framewise displacement during diffusion-weighted imaging.

### Association of BMI and reward network metrics

**Fig. 1:**
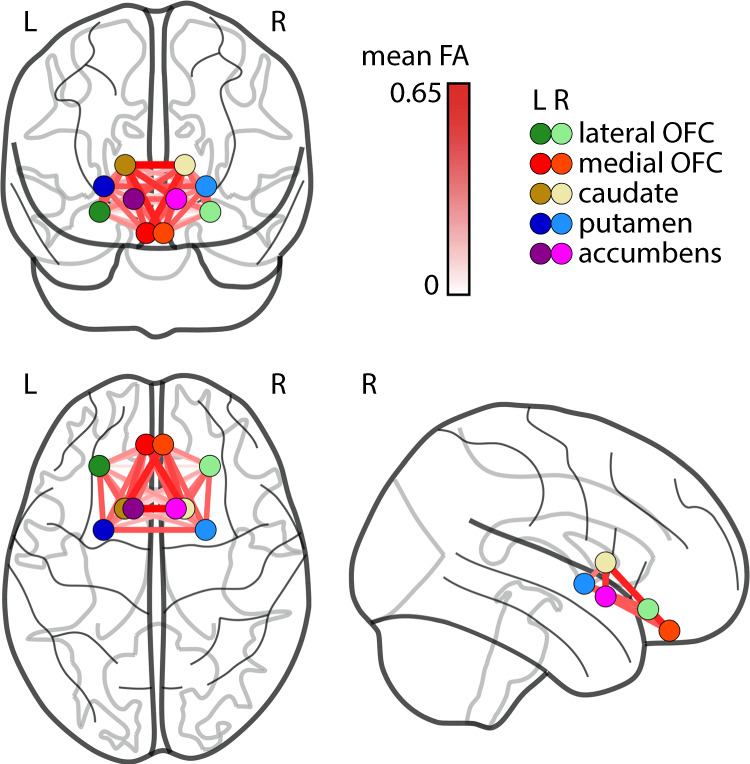
Structural connectivity measured using mean fractional anisotropy (FA) between hubs of the reward network. OFC orbitofrontal cortex, L left hemisphere, R right hemisphere.

In the main linear regression analysis, reconstruction of structural connectivity showed that a higher BMI was associated with lower CS of NOS edge measures in the reward network, after adjusting for age and sex (Bonferroni-corrected, *p* = 0.0042; model 1, Fig. 1 and Table 2). Higher BMI was also associated with lower FA CS, but this effect did not survive Bonferroni-correction (*p* = 0.031). NOS CS remained significantly associated with BMI when additionally correcting for potential confounding effects of head motion, smoking status, depression score, and education (model 2, *p* = 0.015), while FA CS did not. The CC for both FA and NOS was not associated with BMI. When excluding three individuals with highest BMI (marked as moderate outliers), the result for FA (but not NOS) was attenuated and no longer significant.

**Table 2:** Results from the linear regression analysis of BMI and reward network structural connectivity strength (CS) and clustering coefficient (CC) with edge weights fractional anisotropy (FA) and number of streamlines (NOS).

		FA CS	FA CC	NOS CS	NOS CC
Model 1	Adj. *R*^2^ *β* C.I. *p*	0.12 −0.00089 [−0.0016, −0.00008] *0.031*	0.11 −0.02 [−0.043, −0.0024] 0.078	0.12 −0.0025 [−0.004, −0.0008] **0.0042**	0.007 0.31 [−0.09, 0.72] 0.12
Model 2	Adj. *R*^2^ *β* C.I. *p*		–	0.10 −0.0025 [−0.004, −0.0005] *0.015*	–

Given values are adjusted *R*^2^, *β*, 95%—confidence interval (C.I.) of *β* and two-sided *p* value for the effect of BMI (Models 1 and 2). Uncorrected significant *p* values are highlighted in italic, Bonferroni-corrected, significant *p* values are highlighted in bold.

In addition, within-network node-wise measures showed that for NOS, lower CS of the left putamen, and less so, right Nacc were significantly associated with higher BMI (Table 3). In line with the network-wide results, lower FA CS of the bilateral Nacc and putamen, as well as right lateral OFC, were weakly associated with higher BMI (Table 3). When excluding three individuals with highest BMI, node-wise associations of BMI and NOS CS, but not FA, remained significant.

**Table 3:** Results from the linear regression analysis of BMI and node-wise connectivity strength (CS) with edge weights fractional anisotropy (FA) and number of streamlines (NOS).

	Hemisphere	Adj. *R*^2^	*β*	C.I.	*p*
FA CS					
Nacc	Right	0.08	−3.0e−04	[−7.2e−04;−1.3e−04]	*0.05*
	Left	0.06	−2.8e−04	[−6.5e−04; −7.4e−05]	0.12
Caudate	Right	0.01	1.8e−04	[−8.6e−05; 4.1e−04]	0.20
	Left	0	−1.0e−04	[−3.1e−04; 1.1e−04]	0.34
Putamen	Right	0.08	−1.7e−04	[−3.8e−04; 4.9e−05]	0.11
	Left	0.05	−2.5e−04	[−4.7e−04; −2.4e−05]	*0.03*
Medial OFC	Right	0.09	−6.9e−05	[−2.7e−04; 1.4e−04]	0.51
	Left	0.08	−1.4e−04	[−3.6e−04; 7.6e−05]	0.19
Lateral OFC	Right	0.06	−3.5e−04	[−7.0e−04; 3.1e−06]	*0.05*
	Left	0.07	7.9e−05	[−2.7e−04; 4.3e−04]	0.65
NOS CS					
Nacc	Right	0.05	−5.1e−04	[−1.0e−03; 7.1e−06]	*0.05*
	Left	0.06	−2.8e−04	[−7.2e−04; 1.5e−04]	0.19
Caudate	Right	0.05	−6.2e−05	[−6.3e−04; 5.1e−04]	0.83
	Left	0	−4.5e−04	[−1e−03; 1.6e−04]	0.15
Putamen	Right	0.07	−6.6e−04	[−1.4e−03; 1.5e−04]	0.11
	Left	0.09	−1.3e−03	[−2.2e−03; −4.3e−04]	**0.004**
Medial OFC	Right	0.02	−4.2e−04	[−9.2e−04; 2.5e−04]	0.21
	Left	0.06	−4.7e−04	[−1.1e−03; 1.6e−04]	0.14
Lateral OFC	Right	0.04	−3.9e−04	[−9.2e−04; 1.4e−04]	0.15
	Left	0.04	−3.1e−04	[−7.9e−04; 1.5e−04]	0.19

Given are adjusted *R*^2^, *β*, 95%—confidence interval (C.I.) of *β* and two-sided *p* value for the effect of BMI (Model 2, adjusted for age, sex, depression, smoking status, education, head motion during scan). Uncorrected significant *p* values are highlighted in italic, Bonferroni-corrected, significant *p* values are highlighted in bold. Nacc Nucleus Accumbens, OFC orbitofrontal cortex

### Association of genotypes and BMI

**Fig. 2:**
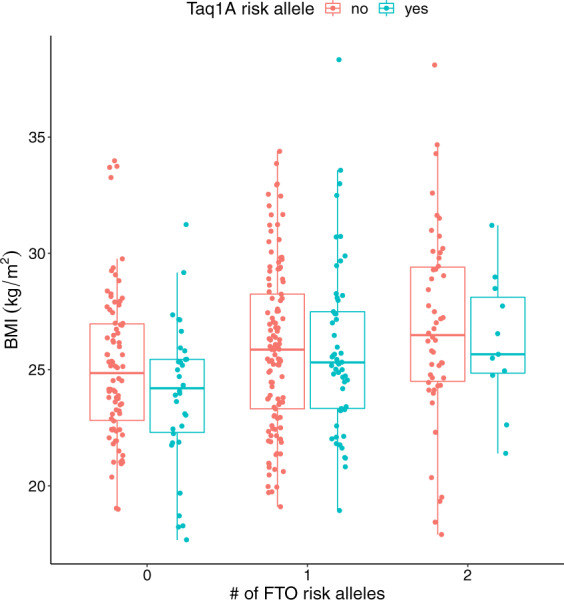
Body mass index (BMI) depends on FTO- and Taq1A genotypes. Individual data points are shown for carriers of 0, 1, or 2 FTO risk alleles (*x*-axis) with no or at least one Taq1A risk allele (red/blue, respectively). Bars within boxes give medians, surrounding boxes show the interquartile range, vertical lines indicate 1.5 times the interquartile range above/below the upper/lower quartile, respectively.

There was no interaction effect of FTO rs1558902 and Taq1A on BMI (adjusted for age and sex, *F* = 1.7, *p* = 0.19). As main effect, we observed a significant increase in BMI with increasing number of FTO rs1558902 risk alleles (adjusted for age and sex, *F* = 6.9, *p* = 0.001 *T*_ATvsTT_ = 2.7, *T*_AAvsTT_ = 3.5, all *p* < 0.001) (Fig. 2). Carrying at least one risk allele in Taq1A rs180047 was not significantly associated with BMI (adjusted for age and sex, *F* = 2.7, *T*_AT/AAvsTT_ = −1.6, *p* = 0.099).

Considering the reward network measures, we did not observe a significant interaction or main effects of FTO and Taq1A genotypes on structural connectivity when adjusting for age and sex, also when adjusting additionally for BMI (Table 4).

**Table 4:** Results from linear regression analysis of BMI, genetic variants, and reward network structural connectivity.

	Adj. *R*^2^	*β*	*C.I.*	*p*	*p* (adj. for BMI)
FA CC					
FTO × Taq1A	0.087			0.96	0.97
FTO_ATvsTT_	0.093	−0.0044	[−0.052; 0.043]	0.86	0.94
FTO_AAvsTT_		0.0044	[−0.058; 0.067]	0.88	0.64
Taq1A		0.024	[−0.033; 0.061]	0.56	0.67
FA CS					
FTO × Taq1A	0.094			0.94	0.92
FTO_ATvsTT_	0.10	0.00035	[−0.0013; 0.002]	0.68	0.48
FTO_AAvsTT_		−0.00046	[−0.0027; 0.0018]	0.68	0.99
Taq1A		0.0005	[−0.0012; 0.0022]	0.55	0.68
NOS CC					
FTO × Taq1A	−0.004			0.90	0.90
FTO_ATvsTT_	0.0007	−0.59	[−1.44; 0.26]	0.17	0.10
FTO_AAvsTT_		−0.36	[−1.48; 0.75]	0.52	0.33
Taq1A		0.23	[−0.61; 1.08]	0.58	0.48
NOS CS					
FTO × Taq1A	0.099			0.67	0.56
FTO_ATvsTT_	0.10	0.00089	[−0.0028; 0.0046]	0.64	0.37
FTO_AAvsTT_		0.00084	[−0.0041; 0.0058]	0.74	0.38
Taq1A		0.0021	[−0.0016; 0.0058]	0.27	0.39

For the FTO × Taq1A interaction, the adjusted *R*^2^ value of the regression model with the interaction and the *p* value of the interaction, derived from comparison with the null model including age, sex, and main genetic effects, are shown. For individual genetic effects, the adjusted *R*^2^ value of the regression null model without the interaction, and the regression coefficients *β* for Taq1A, FTO_ATvsTT_ (0 vs. 1 risk allele), and FTO_AAvsTT_ (0 vs. 2 risk alleles), 95%—confidence intervals (C.I.) of *β* coefficients and two-sided *p* value of the coefficients are given. The column “*p* (adj. for BMI)” indicates results for the null model additionally adjusted for BMI. FA CC: clustering coefficient of the reward network based on fractional anisotropy, FA CS: connectivity strength of the reward network based on fractional anisotropy, NOS CC: clustering coefficient of the reward network based on number of streamlines, NOS CS: connectivity strength of the reward network based on number of streamlines.

*FA CC* clustering coefficient of the reward network based on fractional anisotropy, *FA CS* connectivity strength of the reward network based on fractional anisotropy, *NOS CC* clustering coefficient of the reward network based on number of streamlines, *NOS CS* connectivity strength of the reward network based on number of streamlines.

### Exploratory analyses

Furthermore, we tested for a potential interaction of sex and age on our results. While men had lower FA values on average compared to women, we did not detect a significant interaction of sex and BMI on network strength (model comparison FA: *F* = 0.35, *p* = 53; NOS: *F* = 0.25, *p* = 0.62). We observed a trend for an interaction between BMI and age, indicating stronger negative association of BMI and network strength in younger participants (model comparison, FA: *F* = 1.5, p = 0.22; NOS: *F* = 3.3, *p* = 0.067).

## Discussion

In this cross-sectional analysis of young to middle-aged adults, a higher BMI was significantly associated with lower structural connectivity of the reward network. More specifically, higher BMI predicted less connectivity strength for NOS, and to a lesser degree FA edge weights in the Nacc, putamen and orbitofrontal cortex, even if adjusting for age, sex, education, and other conditions such as depressive symptoms. In addition, while obesity-related SNPs in the FTO gene significantly predicted higher BMI, we did not observe genetic associations with measures of structural connectivity in the reward network.

Our results of lower reward network connectivity with higher BMI are in line with a previous study that examined obesity-related differences in measures of white matter structural coherence. Using the same graph-based approach, Marqués-Iturria et al. reported lower CS and network clustering of the striatum and orbitofrontal cortex in 31 obese participants compared to 32 lean controls [27]. In contrast, [47] reported higher structural connectivity of the bilateral putamen in obese and overweight participants compared to normal-weight controls. This study also investigated sex-by-weight status interactions and found that among individuals with overweight and obesity women had stronger connectivity of reward network regions than men. Our exploratory analyses showed similar results, indicating less CS in men compared to women, and a potential moderation by age. While we chose the same definition of structural connectivity as Marqués-Itturia, Gupta et al. derived their connectivity values from the number of connections and used different graph metrics. This might be an explanation for the diverging results. Yet, analyses in larger samples support our finding and showed that higher BMI was associated with lower FA within white matter tracts throughout the brain, including connections between reward network regions [30, 31, 48]. Longitudinal studies suggest that, at least in older adults, these differences may reflect damage due to the metabolic consequences of vascular risk factors [49, 50]. Regardless of its origin, lower structural connectivity in the reward network might underlie certain behavioral traits observed in obesity, such as aberrant processing of food-related reward stimuli [12, 51].

Considering genetic effects, we replicated the known association of FTO risk alleles and higher BMI, while carrying the Taq1A risk allele was not associated with higher BMI in our cohort.

We did not find an association between the genetic variants in FTO and near DRD2 on reward network structural connectivity in this population-based cohort.

Previous studies indicate that the link between obesity-related genetic variation, obesity, and white matter microstructure is complex: while in a large cohort of Mexican-American families, BMI and white matter microstructure in different fiber tracts were genetically correlated [52], few studies have shown associations of single obesity-related variants and white matter microstructure. Dennis and colleagues reported that among 15 obesity-associated genetic variants, a variant in NEGR1 had the strongest effect on fractional anisotropy in the corona radiata in a sample of ~500 young, mainly normal-weight adults [53]. Further, cumulative effects of all considered obesity-associated genotypes predicted white matter microstructure in arcuate fasciculus, fornix, and inferior frontal-occipital fasciculus. Olivo et al. showed that BMI and FTO interacted on structural connectivity of striatal-frontal tracts in a small sample of young individuals in the normal to overweight weight spectrum [32].

Regarding genetic variants in dopamine D2/D3 receptors (DRD2), which may relate to obesity via differences in reward processing [54, 55], only one study investigated the link with white matter microstructure and found that a genetic variant in strong linkage disequilibrium with Taq1A correlated with higher structural connectivity of striatal-frontal tracts in a sample of young, normal-weight participants [25]. Taken together, genetic effects of obesity-related variants on structural connectivity may be subtle and dependent on weight group. Along these lines, while the BMI-by-FTO interaction was not significant in our sample, an exploratory analysis in the normal-weight individuals suggested during the revision showed that carrying more FTO risk alleles was associated with higher NOS CS of the reward network. This finding indicates that environmental factors (e.g. metabolic consequences of obesity) might have blurred the link between genetic factors and structural connectivity in our sample, which also included overweight and obese individuals. Moreover, exploratory analyses suggest that age might play a role in this relationship, possibly by increasing the variance in brain measures and thereby making it more difficult to detect subtle genetic effects.

In summary, future studies in more homogenous (i.e. normal-weight and young individuals) and larger samples with longitudinal designs need to further address the underlying (epi-)genetics of reward structural connectivity and obesity.

### Limitations

In this study, we did not investigate the causal relationship between BMI and structural connectivity of the reward network. Furthermore, our sample was not rich in obese participants, which might limit the power of our analyses. Although our sample size was adequate for an imaging study, we might be underpowered to detect subtle effects of single polymorphisms on brain connectivity. Finally, we did not assess epigenetic and environmental factors in this design such as e.g., physical activity, diet, and sleep [56, 57]. Strengths of our study include the well-characterized cohort that enabled us to adjust for important confounders in our analysis, and the high-resolution DWI protocol.

### Conclusions

We here provide evidence that higher BMI is associated with lower reward network structural connectivity. We did not find any contribution of variants in FTO or near DRD2 receptor gene to reward network structural connectivity, indicating that the genetic influence of these variants is small. Future research should investigate the behavioral implications of structural connectivity differences in the fronto-striatal network and incorporate larger sample sizes with longitudinal designs in order to gain further insight into the genetic determinants of obesity in the brain.

## Data Availability

Along with the code, we provide a synthetic dataset here: https://github.com/fBeyer89/BMI-and-reward-connectivity-in-LIFE-Adult. This data was simulated based on the properties of the original dataset but guarantees anonymity for the participants [46]. Access to the original data may be provided via the LIFE-Adult Study coordination by the authors upon request.

## Code availability

We made the code and a synthetic dataset available here: https://github.com/fBeyer89/BMI-and-reward-connectivity-in-LIFE-Adult. This code allows to reproduce the main results of the paper, yet, more specific investigations may not yield accurate results [46].
